# Expression of Connexins 37/40 and Pannexin 1 in Early Human and *Yotari* (*Dab1^−/−^*) Meninges Development

**DOI:** 10.3390/biomedicines13123088

**Published:** 2025-12-15

**Authors:** Marko Puljiz, Natalija Filipović, Nela Kelam, Anita Racetin, Yu Katsuyama, Katarina Vukojević

**Affiliations:** 1 Department of Surgery, Service of Neurosurgery, Dubrovnik General Hospital, 20000 Dubrovnik, Croatia; puljiz7@gmail.com; 2 Department of Anatomy, Histology and Embryology, University of Split School of Medicine, Šoltanska 2A, 21000 Split, Croatia; natalija.filipovic@mefst.hr (N.F.); nkelam@mefst.hr (N.K.); amuic@mefst.hr (A.R.); 3 Center for Translational Research in Biomedicine, University of Split School of Medicine, Šoltanska 2A, 21000 Split, Croatia; 4 Department of Anatomy, Shiga University of Medical Science, Otsu 520-2192, Japan; kats@belle.shiga-med.ac.jp; 5 Mediterranean Institute for Life Sciences (MedILS), University of Split, Meštrovićevo Šetalište 45, 21000 Split, Croatia

**Keywords:** Cx37, Cx40, *Dab1^−/−^*, meninges development, Panx1, *yotari*

## Abstract

**Background/Objectives**: The meninges, the protective membranes covering the central nervous system, undergo complex developmental processes that are critical for CNS integrity and function. Connexin 37 (Cx37) and 40 (Cx40), members of the connexin family of gap junction proteins, have been implicated in various physiological and pathological processes. They play a critical role in cell–cell communication. The aim of our study was to investigate the expression of connexins Cx37, Cx40, and Panx1 in the meninges of both human and murine models (*yotari* and wild type) at the 6th week/E13.5 and 8th week/E15.5 of developmental stages. **Methods**: Human embryonic tissues (6th–8th week, n = 4 for the 6th week and n = 4 for the 8th week) and mouse embryos (*yotari Dab1^−^/^−^* and wild type, E13.5–E15.5) were collected and fixed in 4% paraformaldehyde. Paraffin sections were stained for Cx37, Cx40, and Panx1 using immunofluorescence. Images were analyzed in ImageJ, and statistical comparisons were performed using one-way ANOVA with Tukey’s post hoc test (*p* < 0.05). **Results**: Cx37 was consistently expressed across all developmental stages, with the highest threshold area percentage observed at E13.5 and E15.5 in murine leptomeninges, demonstrating statistically significant differences compared to controls (*p* < 0.05) and notably from corresponding human stages (*p* < 0.001). Strong Cx37 staining intensity at E13.5 was noted in both wild-type and *yot* mice, while human leptomeninges displayed mild staining at the 6th week of development. In contrast, both human and murine pachymeninges exhibited moderate Cx37 expression. Additionally, the expression of Cx37 in wild-type mice surpassed that of human samples at both E13.5 and E15.5 stages (*p* < 0.01 and *p* < 0.001, respectively). For the developing dura mater, Cx37 expression peaked at E15.5 in *yot* mice, significantly different from both wild-type and human dura mater (*p* < 0.01 and *p* < 0.05). Cx40 expression was highest in the leptomeninges at E15.5. Panx1 was similarly expressed across stages, with the highest threshold area percent observed in wild-type leptomeninges and pachymeninges at E15.5, showing significant differences compared to *yot* mice and human samples (*p* < 0.05). Both leptomeninges and pachymeninges exhibited mild Panx1 staining at E13.5, while stronger staining was observed at E15.5 in murine samples, contrasting with mild intensity in human counterparts. **Conclusions**: These findings highlight the implications of Dab1 deficiency for the expression of gap junction proteins during meninges development, implicating their importance in intercellular communication that is essential for normal meningeal and neurodevelopmental processes.

## 1. Introduction

Often clinically and experimentally overlooked, the meninges—Greek for membranes or envelopes—are essential for brain development, nourishment, and protection. The meninges are layers of membranous tissue that surround the central nervous system (CNS) [1]. There is a growing body of evidence that the cranial meninges, considering their position between the skull and the brain, are instrumental in many embryological events, such as cortical stratification, glial cell differentiation, apoptosis, angiogenesis, and even calvarial formation [2]. The meninges are divided into the pachymeninges or dura mater and the leptomeninges, further divided into the arachnoid mater and the pia mater. There is a considerable difference in their embryological origins, structures, and functions. Paraxial mesenchyme is the origin of the truncal meninges and the meninges of the caudal head regions, while meninges located in the skull regions anterior to the midbrain are of neural crest cell origin [3]. Our understanding of meningeal embryology is still insufficient. However, a myriad of animal models, especially mouse mutants, have demonstrated the importance of the role the meninges play in normal brain and skull development [1,4]. Namely, for skull development, they are involved in the progression of calvarial osteogenesis, while for brain development, they are involved in cell survival, cell migration, generation of neurons from progenitors, and vascularization [1]. On the other hand, the meninges are also crucial for postnatal life as they harbour stem cells, which are of importance for the adult brain, too [5].

For proper intercellular signalling, meningeal cells are connected by gap junctions and hemichannels. Gap junctions are crucial in developing multicellular organisms, mediating cell signalling, differentiation, growth, and death [6]. Communication between the cytoplasm and extracellular environment, on the other hand, is dependent upon hemichannels (HCs) [7]. Both GJs (consisting of two HCs) and HCs consist of connexins (Cxs), which are their core proteins and channel-forming constituents [7]. Several Cxs isoforms have been found expressed in the brain: Cx30, Cx32, Cx36, Cx43, and Cx45 [8,9]. It has been found that neurons primarily express Cx36 and Cx45 [10,11].

Cx37 is known to participate in cell–cell communication through gap junctions, facilitating the exchange of ions and small molecules. It is predominantly expressed in various tissues and has been implicated in the regulation of blood vessel function, modulation of inflammatory responses, and potentially in neural development. During early development, Cx37 is found in the neural crest derivatives that give rise to meningeal cells. Its expression begins around E9 in mice, coinciding with the initial stages of meningeal differentiation. Cx37 expression is tightly regulated by developmental cues, including signalling pathways that guide neural crest cell migration and differentiation.

Cx40 is integral to the formation and maintenance of gap junctions, which enable direct communication between adjacent cells and are expressed by meningeal progenitor cells originating from the neural crest. Understanding the function of Cx40 in meningeal development is vital for unearthing the pathways that govern brain circuit organization and function. Early expression of Cx40 has been observed around E9 in murine models, reflecting its involvement in initial meningeal embryogenesis. Cx40 is known to be influenced by various signalling pathways, including those activated by growth factors that guide the collective behaviour of meningeal progenitors.

Pannexin 1 (Panx1) is a type of channel protein that plays a significant role in cellular communication, inflammation, neurodevelopment, and barrier integrity. Panx1 is involved in the processes that govern neurogenesis and the integration of neurons with surrounding meningeal tissue. The signalling pathways activated by Panx1 may influence the proliferation, differentiation, and survival of neural progenitor cells.

Additionally, mammalian brain development is dependent on extracellular matrix glycoprotein–Reelin [12]. It is instrumental in embryonic neural cell migration and stratification, as well as in postnatal and adult neurotransmission, synaptic plasticity, and memory formation and consolidation. The process of reelin signalling requires the phosphorylation of the Disabled-1 (Dab1) protein, which is an intracellular adaptor in the reelin signalling pathway. Dab1 is phosphorylated upon activation by Reelin through its receptors, leading to downstream signalling cascades involving key effectors such as Src family kinases, PI3K, Akt, and small GTPases like Rac1 and Rap1. These signalling molecules are known to regulate cytoskeletal dynamics, membrane trafficking, and cell motility, processes that are also essential for the localization and function of gap junction proteins like connexins Cx37 and Cx40, and pannexin Panx1. The significance of Disabled-1 (Dab1) phosphorylation in neuronal migration was recognized through observations of the phenotypic similarities between *Dab1* mutant mice and Reeler (*Reelin^−/−^*) mice. *Yotari* mice, which carry a spontaneous autosomal recessive mutation in the *Dab1* gene, exhibit neurological defects, including an unstable gait, tremors, and premature death, resembling the phenotype of *Reeler* mice [13]. Moreover, Reelin–Dab1 signalling has documented roles in neural crest cell migration and cortical neuron positioning, developmental processes that depend heavily on spatially coordinated intercellular communication, a function largely mediated by the gap junction and pannexin channels [14]. While direct links between Dab1 and connexin regulation remain to be fully elucidated, the shared involvement of these signalling pathways in cytoskeletal remodelling and cellular connectivity provides a compelling mechanistic basis to hypothesize that Dab1 deficiency could disrupt connexin expression and function during meningeal development [14,15]. Hence, the Dab1^−/−^ model offers a valuable system to investigate this putative regulatory axis, addressing significant gaps in understanding how Reelin–Dab1 signalling may orchestrate gap junction-mediated neurodevelopmental processes. Therefore, the aim of this study was to investigate the expression pattern of Cx37, Cx40, and Pnx1 and their significance in cellular communication during the early stages of meningeal development of *yotari* mice as well as in the corresponding stages of human meningeal development. Spatial and temporal dynamics of Cx37, Cx40 and Pnx1 expression in meninges development might indicate their implications in CNS health.

## 2. Materials and Methods

### 2.1. Human Tissue Collection and Preparation

Human conceptuses were obtained from the Department of Gynaecology and Obstetrics and the Department of Pathology, with approval from the Ethical Committee of the School of Medicine, University of Split, following the Helsinki Declaration. Poorly preserved samples were discarded. The age of the conceptuses was determined using the Carnegie Stages [16]. According to Carnegie Stages, the chosen stages are the closest to corresponding murine development that will be used for cross-species comparison (human day 44–6th week and day 55–8th week of development vs. murine E13.5–E15.5) [17,18]. A total of 8 normal human conceptuses from the 6th and 8th week of development (Table 1) were collected from the University Hospital in Split following spontaneous abortions or tubal pregnancies. However, all human conceptuses had a normal karyotype (Table 1). The cranial parts of conceptuses were fixed in 4% paraformaldehyde (PFA) in phosphate-buffered saline (PBS), paraffin embedded, cut in a transverse plane (5 µm), and mounted on glass slides. For this analysis only, cranial parts anterior to the midbrain were used.

### 2.2. Animal Model

In this study, *yotari* (*Dab1*^−/−^) and wild-type (C57BL/6N) mice were used. Animal use was approved by the Guidelines for the Care and Use of Laboratory Animals at the Shiga University of Medical Science. The mice were kept in polycarbonate cages in a climate-controlled room (23 ± 2 °C) with a 12 h dark/light cycle and had ad libitum access to food and water. Gravid mice were anesthetized with pentobarbital and underwent transcardial perfusion with PBS at pH 7.2, followed by 4% paraformaldehyde in 0.1 M PBS. Embryos were collected on embryonic days 13.5 (E13.5) and 15.5 (E15.5). The embryos were fixed overnight in 4% paraformaldehyde in 0.1 M PBS for subsequent histological analyses, including hematoxylin-eosin staining and immunofluorescence (IF) staining. For genotyping, the following PCR primers were employed: *yotari*—GCCCTTCAGCATCACCATGCT and CAGTGAGTACATATTGTGTGAGTTCC; wild type—GCCCTTCAGCATCACCATGCT and CCTTGTTTCTTTGCTTTAAGGCTGT [19,20].

### 2.3. Immunohistochemistry and Microscopy

The histological head sections were first deparaffinized using xylene and then rehydrated with water–ethanol solutions. After these steps, the sections were heated in a steam cooker in a citrate buffer (pH 6.0) for 30 min. Once cooled to room temperature, a protein-blocking solution (ab64226, Abcam, Cambridge, UK) was applied for 20 min. The sections were then incubated overnight with primary antibodies (Anti-Cx37/GJA4, ab181701, host rabbit, dilution 1:200, Abcam, Cambridge, UK; Anti-Cx40/GJA5, ab213688, host rabbit, dilution 1:100, Abcam, Cambridge, UK; and Anti-pannexin 1/PANX1, ABN242, host rabbit, dilution 1:150, Merck KGaA, Darmstadt, Germany) in a humid chamber. Following a wash with phosphate-buffered saline, the secondary antibody (AlexaFluor^®^488 Donkey Anti-Rabbit IgG, 711-545-152, Jackson Immuno Research Laboratories Inc., West Groove, PA, USA) was applied for one hour. DAPI (4′,6-diamidino-2-phenylindole) staining was used to visualize the nuclei. Finally, the slides were allowed to air dry and were then covered with a coverslip using Immu-Mount (Shandon, Pittsburgh, PA, USA). To reduce irrelevant background signals, isotype-matched controls and secondary-only samples were employed. Isotype-matched controls involved substituting the primary antibody with one of the same isotypes that do not target the intended protein, which helped reveal any non-specific binding (Figure S1). Secondary-only samples, which excluded the primary antibody, enabled the identification of non-specific interactions of the secondary antibody. These controls confirmed that the signals detected were specific to the target protein rather than resulting from background noise, like leftover paraffin. Sections stained with haematoxylin and eosin were examined using a light microscope (Olympus BX40, Tokyo, Japan) equipped with an Olympus 27 camera (Tokyo, Japan) for image capture. For immunofluorescence imaging, a fluorescence microscope (Olympus BX51, Tokyo, Japan) paired with a Nikon DS-Ri1 camera (Nikon, Tokyo, Japan) was utilized.

### 2.4. Statistical Analysis

Image analysis was performed using ImageJ software (Version 1.53k, National Institutes of Health, Bethesda, MD, USA) and Adobe Photoshop (Adobe, San Jose, CA, USA). The quantitative analysis was performed under blinded conditions. Ten distinct, non-overlapping visual fields of the human and mouse embryonic samples were captured randomly at 40× magnification, maintaining consistent camera settings. Green staining indicated the positive expression of Cx37, Cx40, and Panx1. A median filter subtraction and colour thresholding technique were employed to quantify the section area occupied by the positive signal, providing a quantitative assessment of immunoreactivity as we described previously [21,22,23]. Briefly, colour thresholding was conducted in the RGB colour space by manually setting consistent threshold ranges for each colour channel (specifically, the R, G, and B values) based on preliminary optimization to accurately isolate the signal of interest across all analyzed images. These threshold settings were kept constant for all samples to ensure comparability. Manual thresholding was chosen to better accommodate variations in staining intensity and background across images, providing more precise delineation of the positive areas. The thresholded regions were then quantified as ‘threshold area percentage,’ representing the fraction of the image area exceeding the set thresholds. Statistical data analyses were conducted using GraphPad Software (GraphPad Prism v.8, La Jolla, CA, USA), with a significance threshold set at *p* < 0.05. We used 4 biological replicates for each group and 5 technical replicates per different antibody staining from each embryo. Shapiro–Wilk test was performed on each data group, and the results showed that the data were normally distributed. To compare immunoexpression and identify significant differences between groups, a one-way ANOVA test followed by post hoc Tukey’s test was applied. Data were presented as mean ± SD.

### 2.5. Semi-Quantitative Assessment of Staining Intensity

Semi-quantitative assessment of staining intensity was performed to evaluate the expression levels of selected markers. The staining intensity captured in digital images was categorized into four ordinal levels: no reactivity (−), mild reactivity (+), moderate reactivity (++), and strong reactivity (+++) (Table 2). Four independent researchers (M.P., N.K., A.R., and K.V), blinded to experimental groups, visually scored the images to ensure objectivity and to account for inter-observer variability. This scoring was complemented by the use of ImageJ software (National Institutes of Health, Bethesda, MD, USA), which facilitated consistent analysis of staining intensities by providing quantitative pixel intensity measurements. The combination of human visual scoring and software-assisted quantification enabled a robust semi-quantitative evaluation of immunoreactivity, balancing subjective appraisal with objective data extraction to support reproducibility and reliability in interpreting differential marker expression across samples.

## 3. Results

### 3.1. Development of Meninges

**Figure 1:**
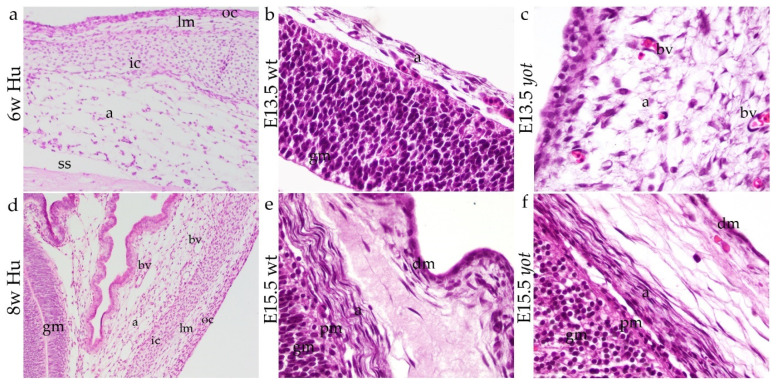
Frontal section of human meninges in the 6th week of development, magnification ×10 (**a**), E13.5 wt mice magnification ×20 (**b**), E13.5 *yot* magnification ×20 (**c**), human meninges in the 8th week of development magnification ×10 (**d**), E15.5 wt mice magnification ×20 (**e**), and E15.5 *yot* magnification ×20 (**f**); a—arachnoid, ic—inner mesenchymal condensation, oc—outer mesenchymal condensation, ss—subarachnoidal spaces, lm—loose mesenchyme, gm—grey mater, pm—pia mater, bv—blood vessels, and dm—dura mater. Hematoxylin-eosin (H&E) staining.

The development of the human meninges occurs during the embryonic period and is particularly intensive between the 6th and 8th weeks. In this period, the neural tube is fully closed, and the lateral plate mesoderm and paraxial mesoderm begin to differentiate into the structures that will form the meninges. The mesodermal cells condense around the developing nervous system, leading to the formation of the innermost layer, leptomeninges (pia mater and arachnoid mater) (Figure 1a). At the outermost layer, dura mater originating from the mesoderm also begins to develop during this period (Figure 1a). However, these two meninges layers become more distinct and more apparent in the 8th week of development (Figure 1b). The space between the pia mater and arachnoid layer begins to form the subarachnoid space, which will later contain cerebrospinal fluid. Meningeal blood supply is established during this week. The pia mater adheres closely to the surface of the brain, while the arachnoid and dura mater layers are well-defined, providing robust protection to the CNS. This arrangement offers the foundation for the further growth and development of the central nervous system in conjunction with the protective layers.

### 3.2. Immunofluorescence Staining with Cx37 Marker

**Figure 2:**
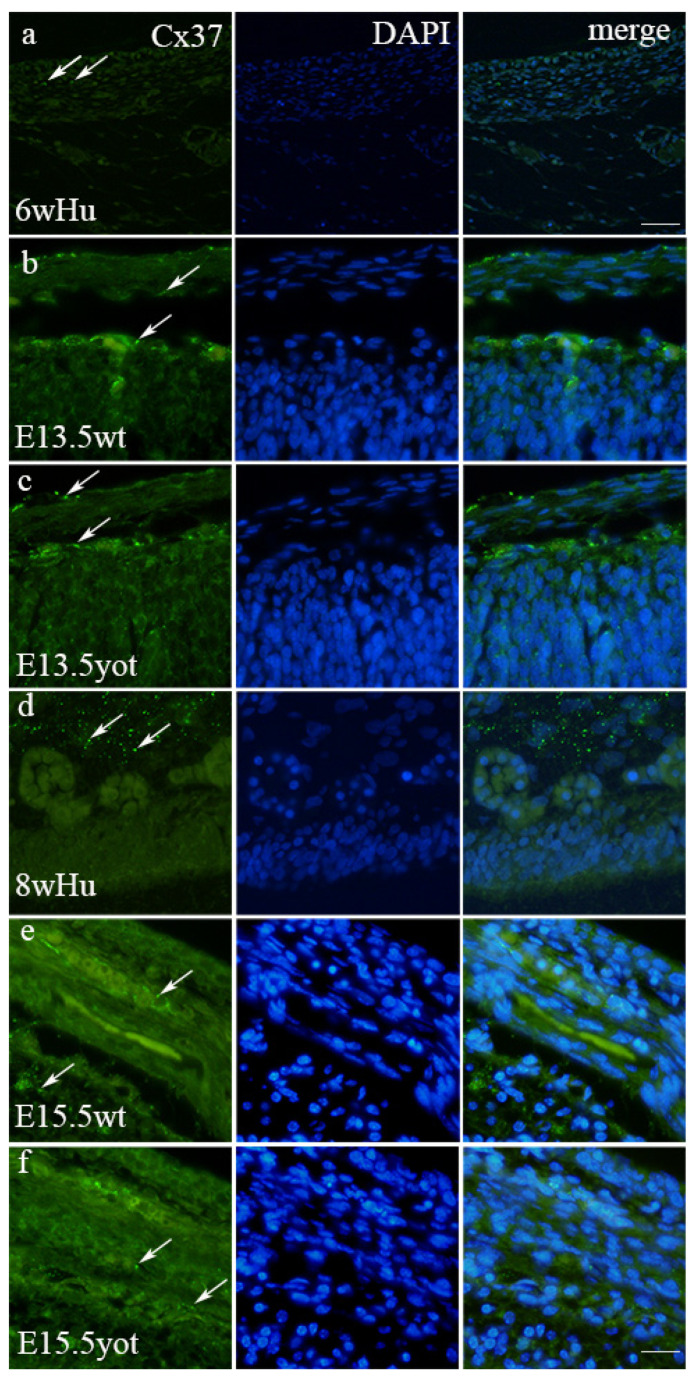
Cx37-positive cells (arrows) in the normal human (Hu), wild-type (wt), and *yotari* (*yot*) mice in meninges at 6th week (6w)/E13.5 and 8th week (8w)/E15.5 developmental stage. Scale bar 25 μm (panel (**a**)) and 10 μm (panels (**b**–**f**)).

**Figure 3:**
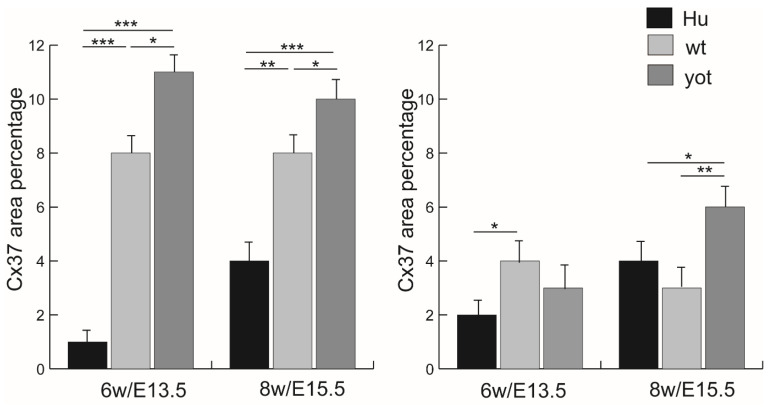
Threshold area percent of Cx37 in the normal human (Hu), wild-type (wt), and *yotari* (*yot*) mice in leptomeninges (**left panel**) and the normal human (Hu), wild-type (wt), and *yotari* (*yot*) in the pachymeninges (**right panel**) at 6th week/E13.5 and 8th week/E15.5 developmental stage. Data were shown as mean ± SD. Statistically significant differences are * *p* ˂ 0.05, ** *p* ˂ 0.01, and *** *p* ˂ 0.001 (ANOVA and Tukey post hoc test).

The expression pattern of Cx37 in humans reveals distinct differences at 6 weeks compared to 8 weeks of development (Figure 2a,d). In the 6th week, Cx37 demonstrates mild intensity in the leptomeninges, contrasting sharply with the strong expression observed at 8 weeks (Table 2). This pattern is paralleled in mice, where at E13.5, the yotari genotype exhibits significant Cx37 expression (11% threshold area percent) in the leptomeninges, and at E15.5, retains high expression at 10% (Supplementary Table S1). Notably, these levels are statistically significantly different from controls (*p* < 0.05), and markedly so, compared to the corresponding human developmental stages (*p* < 0.001), as shown in Figure 3. The intensity of Cx37 staining is strong at E13.5 for both wild-type and yot mice but remains mild in humans at 6 weeks. In the pachymeninges (dura mater), moderate expression is evident in both species (Table 2). At the 8th week of development, human leptomeninges and dura mater show strong intensity, while corresponding E15.5 stages in both wild-type and yot mice reflect only moderate intensity (Table 2). Furthermore, the threshold area percent of Cx37 is higher in wild-type mice at both E13.5 and E15.5 compared to the respective human stages (*p* < 0.01 and *p* < 0.001). The peak expression in the developing dura mater is seen at E15.5 yot (8%), again surpassing wild-type and human values (*p* < 0.01 and *p* < 0.05). Interestingly, the threshold area percent of Cx37 in the wild type is highest at E13.5 when compared to E13.5 yot and human 6th week dura mater, with this difference being statistically significant (*p* < 0.05) (Figure 3).

### 3.3. Immunofluorescence Staining with Cx40 Marker

**Figure 4:**
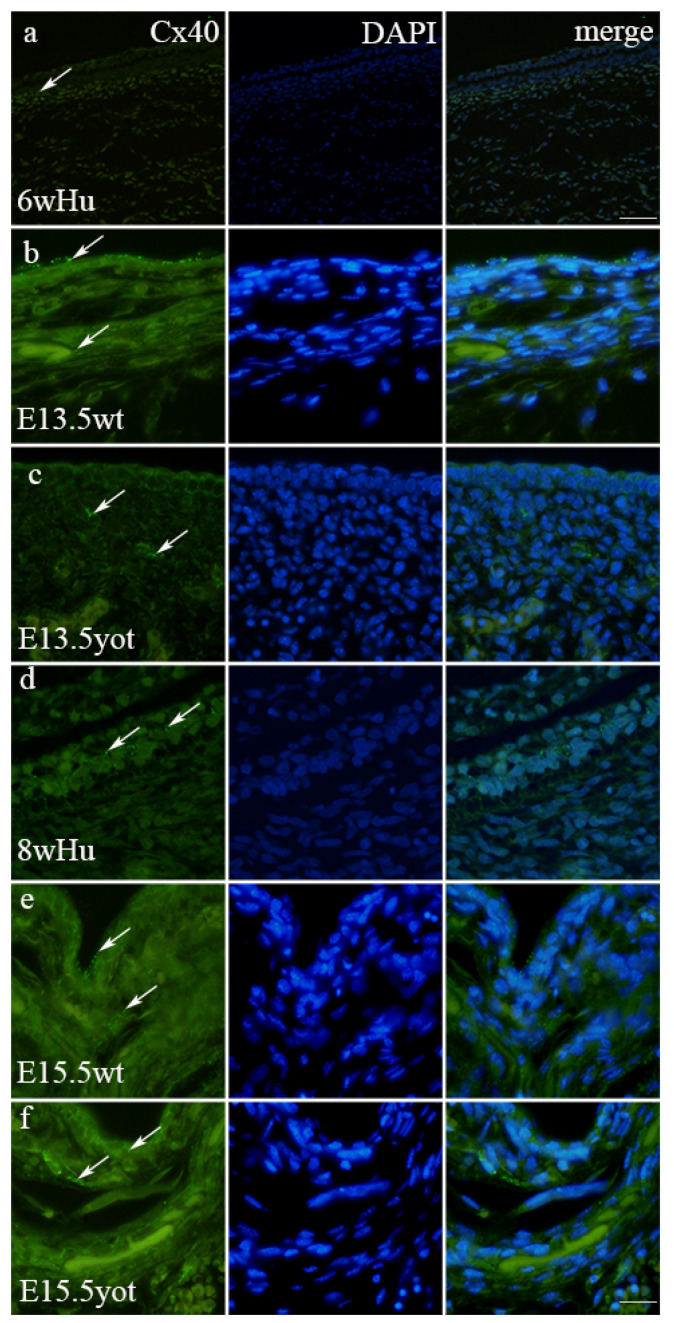
Cx40-positive cells (arrows) in the normal human (Hu), wild-type (wt), and *yotari* (*yot*) mice in meninges at 6th week (6w)/E13.5 and 8th week (8w)/E15.5 developmental stage. Scale bar 25 μm (panel (**a**)) and 10 μm (panels (**b**–**f**)).

**Figure 5:**
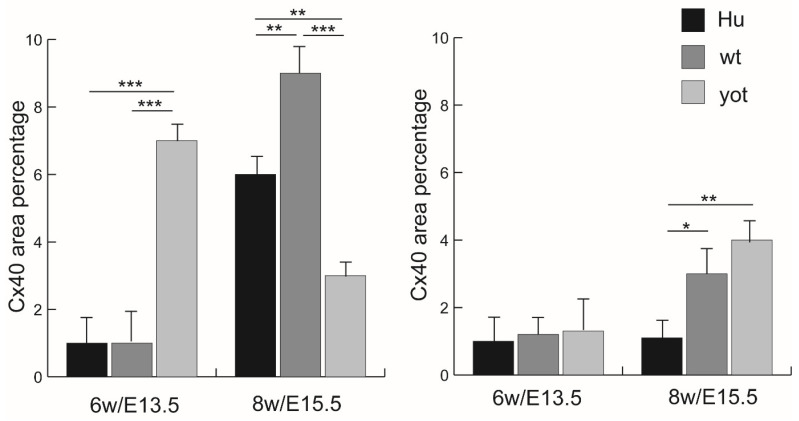
Threshold area percent of Cx40 in the normal human (Hu), wild-type (wt), and *yotari* (*yot*) mice in leptomeninges (**left panel**) and the normal human (Hu), wild-type (wt), and *yotari* (*yot*) in the pachymeninges (**right panel**) at 6th week/E13.5 and 8th week/E15.5 developmental stage. Data were shown as mean ± SD. Statistically significant differences are * *p* ˂ 0.05, ** *p* ˂ 0.01, and *** *p* ˂ 0.001 (ANOVA and Tukey post hoc test).

The expression pattern of Cx40 in humans increases in leptomeninges from 6 weeks toward 8 weeks of development (Figure 4 and Figure 5). In the 6th week, Cx40 demonstrates mild intensity in the leptomeninges, contrasting moderate expression observed at 8 weeks (Table 2), while Cx40 expression in pachymeninges remains mild at both time points (Table 2). Cx40 staining was positive in all developmental stages in both human and mouse meninges (Figure 4a–f). This pattern is paralleled in wt mice, where at E13.5, the wt genotype exhibits 1% of Cx40 in the leptomeninges, and the threshold area percent increases to 9% at E15.5 (*p* < 0.05) (Figure 5) (Supplementary Table S1). On the contrary, in yot mice, Cx40 expression decreased from 7% to 3% from E13.5 to E15.5 5 (*p* < 0.05) (Figure 5). The staining intensity is mild at E13.5 for both wild-type and yot mice in leptomeninges and moderate at E15.5 for both wild-type and yot mice (Table 2). In the pachymeninges (dura mater), mild expression is evident in both species and mice genotype (Table 2). In the pachymeninges, Cx40 expression is very low at the 6th and 8th week of human meningeal development (1%), while in mice, the genotype increases from 1.2% at E13.5 to 4% at E15.5 yot mice (Figure 5).

The peak expression in the developing dura mater is seen at E15.5 yot (4%), again surpassing wild-type values (*p* < 0.05).

Cx40 staining was also seen in the walls of blood vessels (Figure 4c,d,f).

### 3.4. Immunofluorescence Staining with Panx1 Marker

**Figure 6:**
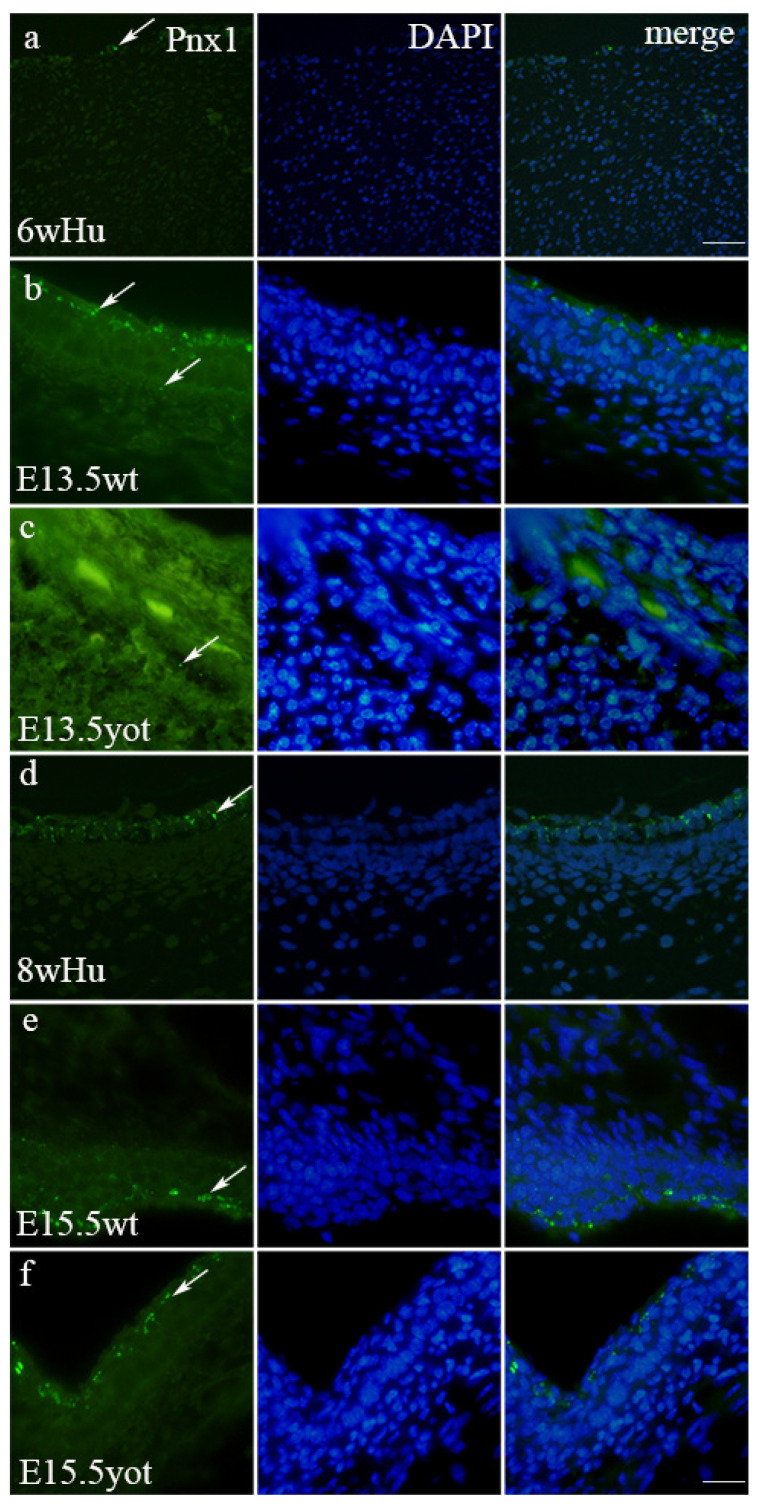
Pnx1-positive cells (arrows) in the normal human (Hu), wild-type (wt), and *yotari* (*yot*) mice in meninges at 6th week (6w)/E13.5 and 8th week (8w)/E15.5 developmental stage. Scale bar 25 μm (panel (**a**)) and 10 μm (panels (**b**–**f**)).

**Figure 7:**
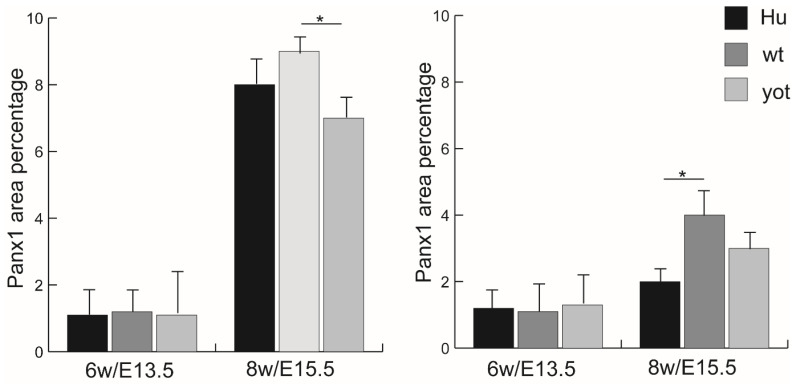
Threshold area percent of Pnx1 in the normal human (Hu), wild-type (wt), and *yotari* (*yot*) mice in leptomeninges (**left panel**) and the normal human (Hu), wild-type (wt), and *yotari* (*yot*) in the pachymeninges (**right panel**) at 6th week/E13.5 and 8th week/E15.5 developmental stage. Data were shown as mean ± SD. Statistically significant differences are * *p* ˂ 0.05(ANOVA and Tukey post hoc test).

The expression pattern of Panx1 in humans and mice reveals notable developmental differences (Figure 6a–f). In humans, at 6 weeks (6w), the Panx1 expression increases from 1% to 8% at the 8th week of development (*p* < 0.05) (Figure 7) (Supplementary Table S1). Additionally, Panx1 shows mild staining intensity, while at 8 weeks (8w), the intensity increases significantly in the leptomeninges, contrasting with the mild intensity observed in the 8w human samples (Table 2). In mice, the pattern is similar. At E13.5, both wild-type (wt) and yotari mice exhibit 1% of Panx1 expression in the leptomeninges and at E15.5, 9% and 7% of Panx1 expression in wt and yot mice, respectively (*p* < 0.05) (Figure 7). However, by E15.5, the wt mice demonstrate significant Panx1 expression with a high threshold area percent of 9% in the leptomeninges and 4% in the pachymeninges, revealing statistical differences between the leptomeninges of E15.5 wt and yotari mice (*p* < 0.05). Additionally, a comparison between E15.5 yotari and the 8w human samples highlights key differences (*p* < 0.05). This expression pattern emphasizes the contrasting Panx1 staining intensity: the leptomeninges and pachymeninges of both mouse groups showed strong staining at E15.5, while the human samples maintained mild staining at 8w in pachymeninges (Table 2).

## 4. Discussion

The present study provides new insights into the expression of connexins (Cx37, Cx40) and pannexin 1 (Panx1) during early meningeal development in both human and mouse embryos. By comparing normal and *Dab1*-deficient (*yotari*) mice, we aimed to explore how alterations in cell communication might affect meningeal differentiation and organization. Our findings highlight the potential involvement of these channel proteins in early intercellular signalling events that guide the formation and maturation of the meninges.

The analysis of Cx37 showed marked differences in expression intensity between 6 weeks and 8 weeks of human development. At 6 weeks, Cx37 displayed mild staining in the leptomeninges, which was significantly enhanced by 8 weeks. This timing corresponds with critical stages in neurodevelopment where neurovascular interactions are essential. In mouse models, particularly in the yotari (yot) genotype, Cx37 expression exhibited high levels at E13.5, with a decrease observed by E15.5. This is consistent with the study by Juric et al., who also found increased Cx37 expression in the arachnoid during human spinal cord development [24]. However, in the pia mater, the Cx37 expression was lower. This difference might be explained by the different embryonic origins of spinal cord meninges, derived from paraxial mesenchyme, vs. meninges anterior to the midbrain, derived from neural crest [3]. Additionally, Cx37 was expressed in both studies, mostly around blood vessels in the leptomeninges, suggesting potential involvement in cerebral blood vessel development. Also, our findings suggest a temporal regulation of Cx37 expression that might differ not only between species but also within genotypes, indicating a possible involvement in vascular maturation and CNS integrity. The statistically significant differences in Cx37 expression between human and mouse samples imply that, while both models express Cx37, the regulatory mechanisms may be fundamentally different, potentially affecting developmental outcomes. The greater threshold area percentage observed in mice, notably at E13.5 and E15.5, reinforces the idea that murine models may provide insights into the significance of Cx37 in neural development that may not be directly translatable to humans. The statistically significant differences in threshold area percent between the *yot* mice and control groups underscore the possible influence of genetic factors on Cx37 expression, particularly when compared with human developmental stages. The strong staining intensity at E13.5 in both wild-type and yot mice contrasts markedly with the mild intensity observed in the human leptomeninges at the 6th week of development. This might suggest potential species-specific variations in Cx37 expression patterns, which may be critical during early brain development and suggest a role for Cx37 in the enhanced development of murine meninges. Additionally, the differential expression in pachymeninges, with moderate levels observed in both species but variation in peak expression timings, highlights the importance of spatio-temporal expression patterns. The highest threshold area of Cx37 in the dura mater of wild-type mice at E15.5 signifies a potential adaptive response in the developing meninges, possibly facilitating cerebrospinal fluid (CSF) flow or neuronal protection mechanisms.

The Cx40 expression demonstrates consistent membranous staining across all developmental stages, with peak expression observed at E15.5 in both wild-type and *yot* mice. The expression patterns of Cx40 further support the trend of increasing expression during developmental progression, with a notable shift from mild expression at 6 weeks to moderate expression at 8 weeks in humans. Interestingly, while Cx40 expression in the pachymeninges remained consistently mild, in mouse models, the wild type exhibited a substantial increase in Cx40 from E13.5 to E15.5, contrasting with a decrease in yot mice, suggesting genotype-specific regulatory cohorts in Cx40 activity. Additionally, in our study, Cx40 displayed a granular staining pattern in blood vessels, especially in the endothelium. The discrepancy in Cx40 expression levels between the yot and wild type at E15.5 underscores the functional implications of connexin regulation on the vasculature of the meninges. Moreover, Cx40’s pronounced staining in the walls of blood vessels suggests its involvement in angiogenic processes, which are crucial during the rapid developmental phases of the brain [25]. This might suggest a crucial role for Cx40 in vascular function within the meninges, affecting the maintenance of the blood–brain barrier. In Cx40-deficient animals (*Cx40^−/−^*), Fang et al. observed a severe reduction in limb perfusion relative to wild-type animals, and they exhibited profound and rapid failure of ischemic limb survival [26]. The same effect was observed in animals with a high expression of Cx37, which shows the opposing influence of these two connexins regarding perfusion. This might suggest the involvement of Cx37 and Cx40 in effective cerebral perfusion important for normal CNS development. The significant differences in threshold area percent between *yot* and wild-type mice, particularly at E13.5, indicate not only a divergence in developmental pathways but also the likelihood that Cx40 may play a role in pathological processes associated with the *yot* genotype. The observed decrease in Cx40 staining intensity and threshold area percent from E13.5/6 weeks to the later stages, alongside the developmental differences from human samples, further supports the idea that molecular mechanisms governing meningeal development may differ significantly across species and stages.

Panx1 expression parallels that of Cx37 and Cx40, with high percentages noted in both leptomeninges and pachymeninges during development. There were significant increases in Panx1 observed from 6 weeks to 8 weeks in humans, while showing enhanced expression in mice over the same developmental periods. The higher threshold area percentages observed in murine models indicate a robust involvement of Panx1 in the developing CNS. The contrasting staining intensities between human and mouse samples point to additional layers of complexity in developmental regulation. Additionally, Kirby et al. found that Paxn1 channels control the hemodynamic response to hypoxia by regulating extracellular ATP, as well as the previously known fact that Panx1 channels mediate smooth muscle cell contraction in arteries [27]. The marked differences between genotypes at E15.5, particularly in the leptomeninges, stress the potential regulatory role of Panx1 in maintaining homeostasis in the developing meningeal environment. The transition from the mild staining intensity at E13.5 to the strong intensity at E15.5 and 8 weeks suggests a critical period where Panx1 expression may serve as a mediator of increased intercellular communication and signalling in response to developmental cues. The strong Panx1 staining observed in the leptomeninges and moderate expression in the pachymeninges during the later stages of development in mice implies that Panx1 may play critical roles in cellular communication and signalling during neurodevelopment. The differences between mouse and human samples, particularly highlighting strong staining in mice at E15.5 versus mild staining in 8-week humans, could suggest differential functional roles of Panx1 in neurovascular coupling processes. Notably, the reduced expression in human samples at 8 weeks compared to both mouse genotypes may indicate evolutionary adaptations specific to human CNS development.

Although early human embryonic material is extremely rare, which inherently restricts cohort size, a limitation of our study is the small sample size, with only eight human embryos. Accordingly, our data are descriptive in nature, but we believe the data still remain informative given the scarcity of such samples. Additionally, the quantification of the immunofluorescence signal is performed by measuring the percentage of the area surpassing a defined intensity threshold. This limitation may affect the accuracy of signal quantification, as using a fixed threshold area percentage can overlook subtle variations in fluorescence intensity and background noise, potentially leading to the under- or overestimation of true signal differences. Also, the comparison between human and murine meninges developmental stages (human 6th–8th week vs. murine E13.5–E15.5) is not precise in timing due to species differences in developmental pacing and milestones. However, the chosen timing is the closest as it can be according to Carnegie Stages (human day 44–6th week and day 55–8th week of development vs. murine E13.5–E15.5) [17,18]. We wanted to highlight that cross-species comparisons focus on identifying conserved processes rather than exact chronological equivalence and that evolutionary rationales for comparisons lie in shared developmental patterns and functional dependencies not exact timings.

## 5. Conclusions

Understanding how the Dab1 mutation affects connexin expression could provide insights into the molecular mechanisms underlying neural development and disorders. The yotari mouse model can serve as an important tool to study human conditions associated with altered neuronal migration and connexin function. The different connexin expression profile in yotari mice due to the Dab1 mutation suggests multiple avenues for investigation regarding neurodevelopment, vascular integrity, neuronal signalling, and potential relevance to human neurological diseases. Our study manifests distinct developmental expressions of Cx37, Cx40, and Panx1 in the meninges of humans and mice, pointing to evolutionary and physiological differences in CNS development. These patterns may provide insights into the understanding of meningeal structures in brain fitness. However, further investigations are warranted to elucidate the mechanistic pathways governing these expression patterns and to explore their implications for neurological health and disease.

## Figures and Tables

**Table 1:** The human embryos examined in this research.

Age (Weeks)	CRL (mm)	Carnegie Stage	No.	No. Karyotype
6	14	16	4	2 46, XX; 2 46, XY
8	27	22	4	2 46, XX; 2 46, XY

CRL—crown-rump length.

**Table 2:** Immunoreactivity to specific antibodies in the developing meninges of the human, wild-type, and *yotari* mice during 6th and 8th developmental weeks in humans and corresponding E13.5 and E15.5 stages in mice.

Primary Antibodies’ Immunoreactivity in the Meninges at Two Developmental Periods		Cx37		Cx40		Pnx1	
		Leptomeninges	Pachymeninges	Leptomeninges	Pachymeninges	Leptomeninges	Pachymeninges
6w/E13.5	Hu	−/+	++	−/+	+	+	+
	wt	+++	++	+	+	+	+
	*yot*	+++	++	+	+	+	+
8w/E15.5	Hu	+++	+++	++	+	+++	+
	wt	++	++	++	+	+++	+++
	*yot*	++	++	++	+	+++	+++

Strong reactivity (+++); moderate reactivity (++); mild reactivity (+); and no reactivity (−).

## Data Availability

The data supporting the findings of this study are available from the corresponding author upon reasonable request.

## Supplementary Materials

The following supporting information can be downloaded at: https://www.mdpi.com/article/10.3390/biomedicines13123088/s1, Figure S1: Isotype control images for the normal human (Hu), wild type (wt) and yotari (yot) mice in meninges at 6th week(6w)/E13.5 and 8th week(8w)/E15.5 developmental stage, assessing Cx37 at 8w Hu (a), Cx40 at E13.5 yot (b) and Pnx1 at E15.5 wt (c). Images were taken at ×40 magnification, with a scale bar of 25 μm applied to all images. Table S1: Leptomeninges (L) and pachymeninges (P) mean values (M), lower confidence interval bounds (LCI), and upper confidence interval bounds (UCI) for expression levels of Cx37, Cx40, and Pnx1 proteins across developmental stages. Data are presented separately for leptomeninges and pachymeninges tissues at stages 6w human (Hu), E13.5 wild type (wt), E13.5 yotari (yot), 8w human (Hu), E15.5 wild type (wt), and E15.5 yotari (yot).

## Abbreviations

The following abbreviations are used in this manuscript:

a	Arachnoid
ANOVA	Analysis of variance
ATP	Adenosine triphosphate
bv	Blood vessel
CNS	Central nervous system
CRL	Crown-rump length
CSF	Cerebrospinal fluid
Cx37	Connexin 37
Cx40	Connexin 40
Dab1	Disabled-1
DAPI	4′,6-diamidino-2-phenylindole
dm	Dura mater
E	Embryonic day
GJ	Gap junctions
gm	Gray matter
HE	Hematoxylin eosin
HC	Hemichannels
HU	Human
ic	Inner mesenchymal condensation
lm	Loose mesenchyme
oc	Outer mesenchymal condensation
Panx1	Pannexin1
PBS	Phosphate buffered saline
PCR	Polymerase chain reaction
PFA	Paraformaldehyde
pm	Pia mater
SD	Standard deviation
ss	Subarachnoid space
*yot*	*Yotari*
wt	Wild-type
