# Supplementary material for: Expression of Connexins 37/40 and Pannexin 1 in Early Human and Yotari (Dab1−/−) Meninges Development
Source: Biomedicines. 2025 Dec 15;13(12):3088. doi: 10.3390/biomedicines13123088 (PMC12730412; doi:10.3390/biomedicines13123088)
Supplement: Supplementary file 1 [file biomedicines-13-03088-s001.zip › biomedicines-3978152-supplementary.pdf]

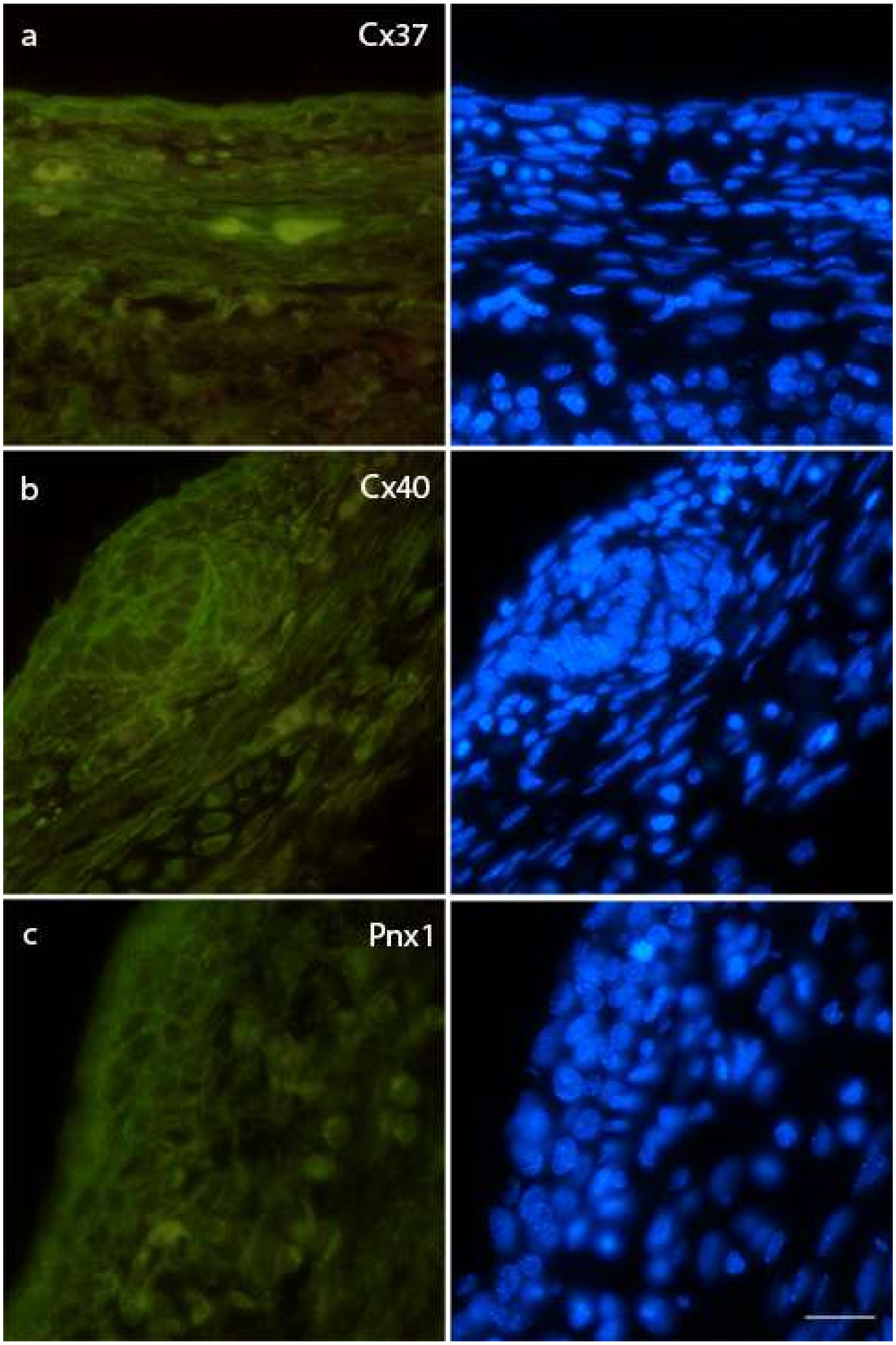

Figure S1. Isotype control images for the normal human (Hu), wild type (wt) and yotari (yot) mice in meninges at 6th week(6w)/E13.5 and 8th week(8w)/E15.5 developmental stage, assessing Cx37 at 8w Hu (a), Cx40 at E13.5 yot (b) and Pnx1 at E15.5 wt (c). Images were taken at ×40 magnification, with a scale bar of 25 μm applied to all images.

Table S1. Leptomeninges (L) and pachymeninges (P) mean values (M), lower confidence interval bounds (LCI), and upper confidence interval bounds (UCI) for expression levels of Cx37, Cx40, and Pnx1 proteins across developmental stages. Data are presented separately for leptomeninges and pachymeninges tissues at stages 6w human (Hu), E13.5 wild type (wt), E13.5 yotari (yot), 8w human (Hu), E15.5 wild type (wt), and E15.5 yotari (yot).

	Cx37						Cx40						Pnx1					
	L			P			L			P			L			P		
stage	M	LCI	UCI	M	LCI	UCI	M	LCI	UCI	M	LCI	UCI	M	LCI	UCI	M	LCI	UCI
6w Hu	1	0.6	1.4	2	1.5	2.6	1	0.1	1.9	1	0.2	1.9	0.8	0.1	1.9	1.1	0.6	1.6
E13.5 wt	8	7.6	8.5	4	3.2	4.7	1	0.1	2.1	1.1	0.6	1.7	1	0.4	1.5	1	0.4	1.6
E13.5 yot	11	10.6	11.7	3	2.2	3.8	7	6.5	7.3	1.2	0.3	2.1	0.9	0.1	2.3	1.2	0.2	2.1
8w Hu	4	3.2	4.8	4	3.3	4.8	6	5.4	6.5	1	0.5	1.5	8	7.2	8.7	2	1.8	2.2
E15.5 wt	8	7.3	8.7	3	2.2	3.8	9	8.2	9.9	3	2.5	3.6	9	8.6	9.3	4	3.4	4.5
E15.5 yot	10	9.4	10.7	6	5.2	6.8	3	2.5	3.3	4	3.5	4.4	7	6.5	7.4	3	2.5	3.4
